# Fused silica optical fibers with graded index nanostructured core

**DOI:** 10.1038/s41598-018-30284-1

**Published:** 2018-08-17

**Authors:** Alicja Anuszkiewicz, Rafal Kasztelanic, Adam Filipkowski, Grzegorz Stepniewski, Tomasz Stefaniuk, Bartlomiej Siwicki, Dariusz Pysz, Mariusz Klimczak, Ryszard Buczynski

**Affiliations:** 1 Glass Department, Institute of Electronic Materials Technology, Wolczynska 133, 01-919 Warsaw, Poland; 2 Faculty of Physics, University of Warsaw, Pasteura 5, 02-093 Warsaw, Poland

## Abstract

The ability to shape the index profile of optical fibers holds the key to fully flexible engineering of their optical properties and future applications. We present a new approach for the development of a graded index fused silica fiber based on core nanostructurization. A graded index core is obtained by means of distribution of two types of subwavelength glass rods. The proposed method allows to obtain arbitrary graded distribution not limited to the circular or any other symmetry, such as in the standard graded index fibers. We have developed a proof of concept fiber with parabolic refractive index core and showed a perfect match between its predicted, designed and measured properties. The fiber has a core composed of 2107 rods of 190 nm of diameter made of either pure fused silica or Ge-doped fused silica with 8.5% mol concentration. The proposed method breaks the limits of standard fabrication approaches used in fused silica fiber technology.

## Introduction

There is a constant need for new types of optical fibers, which ensure higher bandwidth for telecommunication, higher sensitivity for sensors –compared to existing solutions or mid-infrared transmission^1^. Current progress in fiber optics is determined by the performance of Modified Chemical Vapor Deposition (MCVD) and other variations of CVD method of preform fabrication^2^. These technologies limit variety of considered structures to rotational-like symmetry only. Photonic crystal fiber technology allows fiber engineering though modification of cladding properties. This method allows to modify dispersion in broadband range and to break symmetry of the fiber structure^3^. On the other hand, development of graded index core fibers is not feasible with this method. Also a robust integration with standard fibers is challenging due to air-hole structure in the cladding^4^. An alternative form for shaping properties of fibers is modification of internal structure of the core. Development of free-form gradient index fibers is possible with the recently proposed nanostructurization method^5^.

Standard optical fibers with graded-index (GRIN) parabolic-like refractive index profile are multimode and are attractive in telecommunications due to high bandwidth and low modal dispersion^6^. Multimode GRIN fibers with parabolic profile are also considered in mode-division multiplexing mechanism, where each of the guided modes can be addressed individually^7,8^. Recently, normal dispersion multimode GRIN fibers were used to show self-organization of nonlinear waves, where intermodal interactions are mediated by disorder, nonlinearity and dissipation^9^.

Graded index distribution in waveguides is typically achieved with use of ion exchange^10^. In polymer fibers interfacial-gel polymerization^11^ or multistep polymerization technique^12^ is used. For fine grade fused silica fibers only MCVD techniques^13^ can be applied. In this fabrication processes, the diversity of materials and dopant levels of silica glass are very narrow and, in consequence, obtaining of arbitrary refractive index profile^14^ is limited or impossible. Because of these technology limitations, the single-mode silica GRIN optical fibers have never been considered as a dispersion engineered medium for telecommunication, and the properties of such a fibers could not have been foreseen.

Here, we report for the first time on the designing and fabrication of a single-mode fused silica fiber having a solid, homogeneous cladding and a nanostructured graded index core, in which a refractive index has been designed by a layout of stacked silica and Ge-doped silica rods, with a refractive index contrast of Δn = 12 × 10^−3^. We show that with two silica based glasses it is easy to shape not only index distribution profile, but also the dispersion characteristics. With this approach, broadband engineering of modal, polarization and dispersion properties of fibers is feasible and overcomes current limits of fiber technology.

Originally a concept of a nanostructured graded index medium was introduced for the development of microoptical components^15–18^. The nanostructured parabolic graded index microlenses and microlens arrays^15^, elliptical microlenses^16^, axicon microlenses^17^ and anisotropic components^18^ have been developed. Recently the successful development of a first fiber with nanostructured core based on thermally matched silicate soft glasses^5^ has been shown. However in this case the refractive index profile was not controlled since, as was observed, mobility of various ions is different and diffusion between glasses is not predictable. Moreover, application area of silicate fibers is very limited since attenuation of silicate glasses is usually well above 5 dB/m.

The method for nanostructured fiber development considered in this paper is well matched other previously reported methods dedicated to nanostructurization of optical fibers. New methods for integration of nanostructure features of semiconductor and metal into the optical fibers have also been reported. In particular the new methods for polymer fibers with metamaterial structure for subdiffraction imaging and focusing^19^, fibers with semiconducting nanowires for application in optoelectronics^20^, fibers with arrays of dielectric nanowires for nanosensing and photovoltaic applications^21,22^ or fibers with nanospheres for whispering gallery mode resonators^23^ are proposed. Since all these methods are devoted mainly to other type of materials as metals or semiconductors, than our proposed technology (devoted only to glass) they should be treated as complementary, not competitive. A combination of the previously proposed and our techniques can be used to develop types of multimaterial nanostructured fibers.

In this paper we show a method to develop a fused silica fiber with a potentially arbitrary refractive index distribution in the core. Presented analysis of designed and fabricated nGRIN fibers confirms that it is possible to achieve and control refractive index distribution thanks to the nanostructurization method. Because the standard multimode GRIN fibers have a parabolic index profile, therefore the control over the index distribution in nanostructured fibers was done for this typical gradient. Presented results confirm that nanostructurization method allows to design and fabricate structures with arbitrary refractive index distribution using only two different types of glasses, and that it could find application in fabrication of fibers i.e. which produce top-hat beams or Bessel beams. The proposed method can allow to manufacture single mode fibers with dispersion engineered profiles and multimode fibers with engineered group velocity for various modes. More importantly, this method allows development of fibers with non-radial symmetry. Rectangular shaped mode profile fibers are highly demanded for laser ablation applications^24^. Also, polarization properties can be determined, since artificially anisotropic glass core can be constructed with the nanostructured approach. None of fiber types mentioned above can be developed with standard technology for fused silica fiber development. Here we provide evidence, that the nanostructurization method overcomes this limit. The potential and limits of the proposed method are difficult to estimate at the moment, but as any refractive index value and distribution can be obtained, with use of only two base glass materials, it is very promising in optical fiber technology, as well as in other areas of optics. The fibers with arbitrary refractive index distribution can find application in the optical systems for which the shape of the output beam has to be profiled, e.g. Bessel beams, rectangular cross-section, top-hat and vortex beams^16,25^ or degeneration of polarization components of guided mode should be waived^17^. In particular arbitrary refractive index distribution can enable development of various types of fibers, as a few mode fibers, with well-defined and separated propagation constants or fibers with large mode area and broadband low dispersion, which are highly demanded for further high bandwidth networks^8,26^.

The nanostructurization idea was previously applied to design and fabricate borosilicate fibers^5^, while within this article we prove, that this concept works properly for fused silica fibers and that we can design a fiber with specified parameters and develop it strictly according to the design. Fused silica fibers are the most commonly used and spectrum of their application is the largest among all other types of fibers and for large community of users, as telecom, sensors and biomedical sectors their practical use is incomparably greater.

## Results

### Nanostructured GRIN fiber design

**Figure 1:**
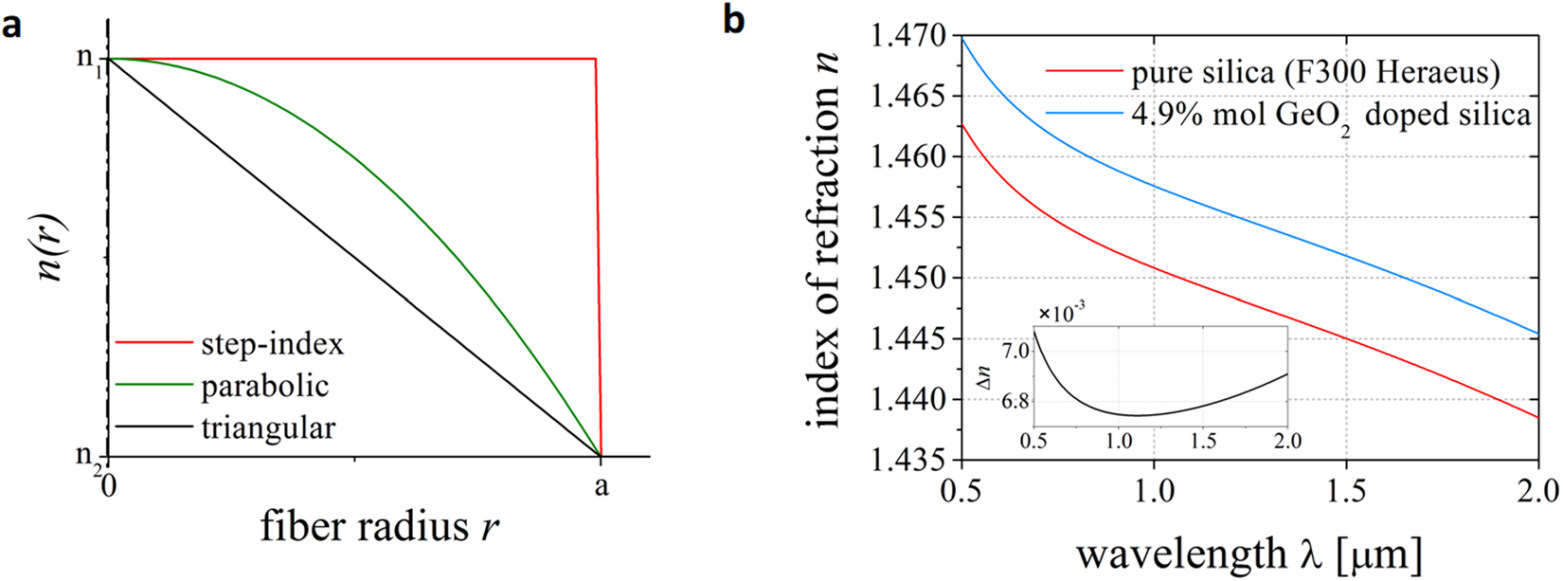
Refractive index profiles and values. (**a**) Radial index profile for the discussed fibers. (**b**) Refractive index for two glasses: pure (red) and 4.9% mol *GeO*_2_ doped (blue) silica and the refractive index difference Δ*n* (inset: black) in a function of wavelength.

Fibers with radial symmetric refractive index distribution are considered. We focused on three selected continuous refractive index profiles presented in Fig. 1a. For all fibers, the radial index profile *n*(*r*) is described by the power-law index formula:

1 $$n(r\le a)={n}_{1}\sqrt{1-2{\rm{\Delta }}{(\frac{r}{a})}^{g}},$$

where Δ is defined as:

2 $${\rm{\Delta }}=({n}_{1}^{2}-{n}_{2}^{2})/(2{n}_{1}^{2}),$$

*n*_1_ and *n*_2_ are refractive indices of the core and the cladding, respectively, *r* is the radial distance, *a* stands for the core radius, and *g* is a profile parameter. By manipulating the value of the *g* parameter it is possible to obtain different refractive index distributions. In particular: a step-index profile fiber for *g* = ∞, a parabolic profile fiber for *g* = 2 and a triangular profile fiber for *g* = 1. For all considered fiber designs, the refractive index outside the core equals *n*_2_:

3 $$n(r > a)={n}_{2}.$$

Other index profiles were recently investigated for chalcogenide nanostructured fibers^27^, i.e. for other *g* values together with spherical index distribution and will not be considered here, as they are similar to parabolic refractive index profile.

Linear simulations of the fiber optical properties were performed using Finite Element Method (FEM). Fused silica glass (vendor designation F300) was assumed for the material with the lowest value of refractive index (*n*_2_). Germanium-doped silica glass was taken for the material with the maximum value of refractive index (*n*_1_). Dispersion of the Ge-doped silica is given by the Sellmeier dispersion equation^28^:

4 $${n}^{2}(\lambda )-1=\sum _{i=1}^{3}\frac{[B{S}_{i}+X(B{G}_{i}-B{S}_{i})]{\lambda }^{2}}{{\lambda }^{2}-{[C{S}_{i}+X(C{G}_{i}-C{S}_{i})]}^{2}},$$

where λ is the wavelength in vacuum, and *BS*_*i*_, *CS*_*i*_, and *BG*_*i*_, *CG*_*i*_ are the Sellmeier coefficients for the *SiO*_2_ and *GeO*_2_ doped glasses, respectively^29^. The value of *X* is the *GeO*_2_ concentration in % mol. The refractive index characteristics for the glasses used in the following simulations (F300 and 4.9% mol Ge-doped silica) and their refractive index difference Δ*n* are shown in Fig. 1b.

**Figure 2:**
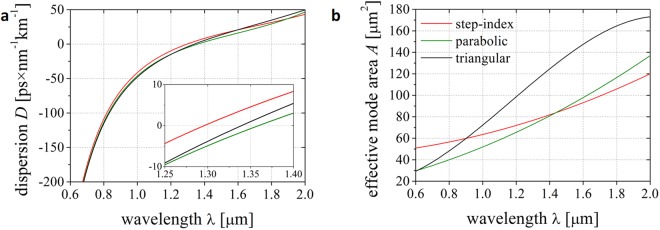
Fundamental mode (**a**) dispersion *D* and (**b**) effective mode area *A* calculated for fibers with different index distribution in the core. Assumed diameter of a core was 9 µm, while the doping level of silica was 4% mol.

Firstly, we examined how dispersion *D* and effective mode area *A* of the fundamental mode change for the particular refractive index distributions. A core diameter of 9 µm was assumed in the simulations, and the *GeO*_2_ concentration in the higher refractive index silica rods was taken at a level of 4% mol. As demonstrated in Fig. 2, the zero dispersion wavelength (ZDW) changes between different fiber designs. In the case of the step-index fiber ZDW is at the wavelength of 1296 nm, for parabolic index profile fiber it is at 1359 nm. ZDW for the triangular index profile fiber is at 1339 nm. The effective mode area *A* of the discussed fibers increases monotonically within the considered wavelength range. For the triangular refractive index profile fiber this increase is faster and less uniform in comparison to other fibers.

**Figure 3:**
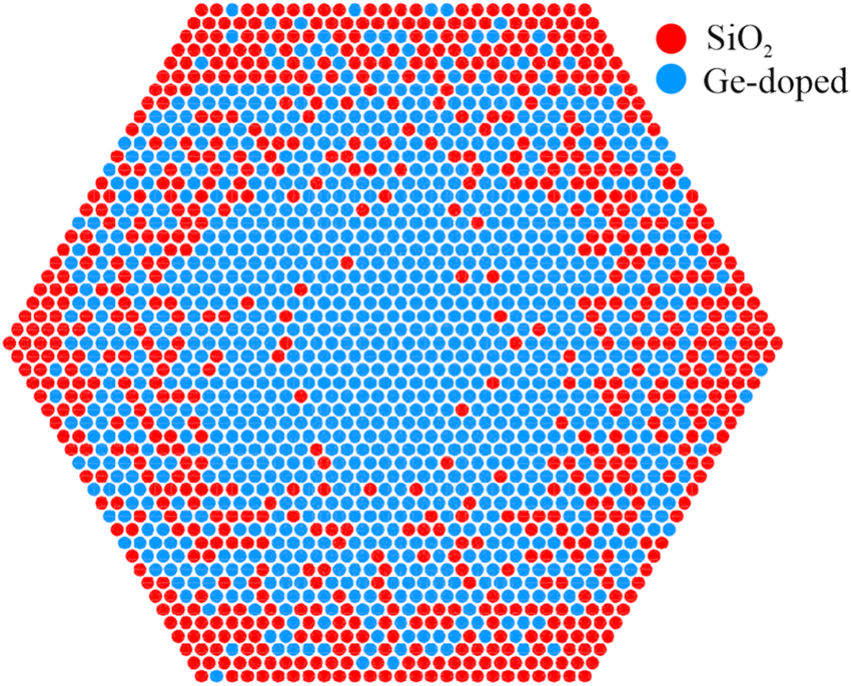
Distribution of glass rods in the core area of a nanostructured fiber with effectively parabolic index profile. The core was composed of 2107 glass rods of pure and Ge-doped silica. The rods are separated for better visibility.

The study carried out so far has been dedicated to the fibers with continuous profile of refractive index in the core. Practical realization of such profiles in fibers is currently limited by technological capabilities of the MCVD methods. In order to work around the limitations of MCVD technology, a radically new approach is proposed here, which is based on an effective medium theory (EMT)^30^. According to EMT, only two types of glass materials with a refractive index contrast, and in a form of glass rods of subwavelength diameter, are sufficient to obtain arbitrary refractive index distribution. The EMT approach leads to a transverse profile of the fiber with a layout of discrete rods (i.e. “pixels”) with subwavelength diameters, as shown in Fig. 3. In the next step of numerical analysis we compared properties of the fibers having pixelated core area, with ideal fibers, having continuous index distribution. We focused on a design with parabolic index distribution, which can be also achieved by standard fabrication methods for GRIN fibers. We used the first order EMT^30^ to change the continuous distribution of the refractive index given by the equation Eq. (1) with *g* = 2 into a binary distribution of subwavelength rods to form which will be further referred to as the “nanostructured” or “nGRIN” fiber. In this approach^15^, the effective dielectric constant of the medium *ε*_*eff*_ depends on the dielectric constants *ε*_1_ and *ε*_2_ of the glasses with the lower and higher refractive index, respectively:

5 $${\varepsilon }_{eff}={\varepsilon }_{1}\frac{{\varepsilon }_{2}(1+2\delta )-2{\varepsilon }_{1}(\delta -1)}{{\varepsilon }_{1}(2-\delta )+{\varepsilon }_{2}(1+\delta )},$$

where *δ* is the relative proportion of the high index material *ε*_2_. The conversion results are presented in Fig. 3.

**Figure 4:**
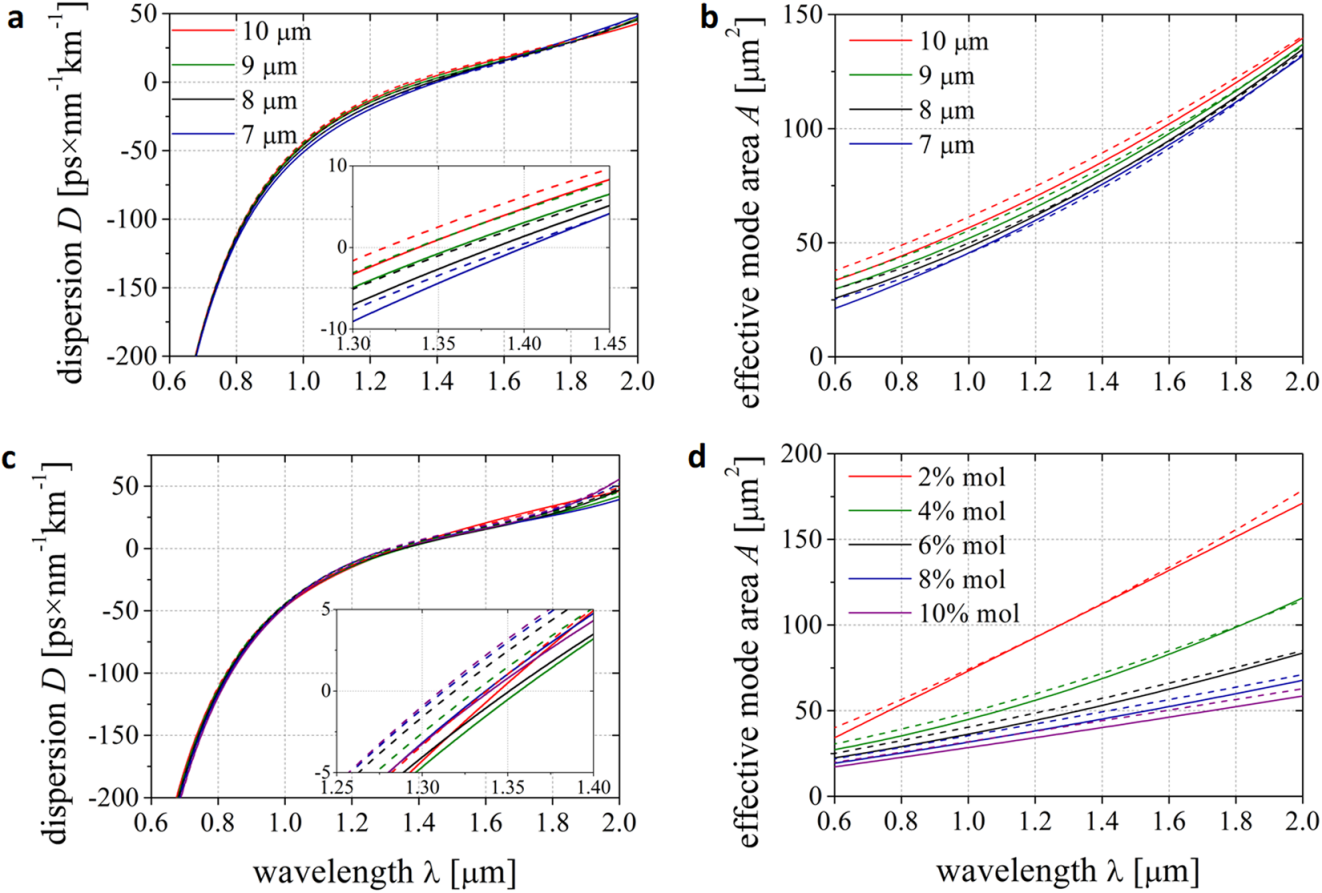
Dispersion *D* and effective mode area *A* calculated for fundamental mode in discussed fibers. (**a**,**b**) Both parameters were investigated for fibers with constant maximum *GeO*_2_ dopant concentration of 4% mol in function of different core sizes, ranging from 7 to 10 μm, and (**c**,**d**) in function of different maximum Ge-dopant concentration, varying from 2 to 10% mol, with constant core size of 9 μm. Solid lines represent parabolic fibers, dashed lines are for nanostructured fibers.

We have calculated the dispersion *D* and effective mode area *A* for a range of wavelengths for the nanostructured fiber and compared the results with *D* and *A* characteristics calculated for the fiber with an ideal parabolic profile of refractive index. Both fibers had fixed maximal Ge-dopant of 4% mol. Additionally, we tested how this parameters depend on the diameter of the core, starting from 7 µm, up to 10 µm (Fig. 4a,b). We also examined how the *GeO*_2_ concentration influences both the dispersion and mode area in fibers with 9 µm core diameter (Fig. 4c,d).

Zero dispersion wavelength shifts towards longer wavelengths as the diameter of the core decreases. The change of the core size from 7 to 10 μm results in ZDW shift of about 75 nm. The discretization of the fiber structure does not change their propagation properties significantly as compared to fiber with assumed ideal refractive index profile. For all nanostructured fibers the dispersion characteristics are lifted up by less than 3 ps × nm^−1^ km^−1^ in comparison to ideal (continuous) graded fibers. The position of ZDW changes within a range of 20 nm. For the fibers with a varied concentration of *GeO*_2_, the ZDW shifts to the shorter wavelengths, as the concentration increases. This change is about 30 nm in concentration range from 2 to 10% mol. The impact of the variation in dopant concentration on the dispersion characteristics is smaller than for core size variations. The discrepancies in the ZDW location between ideal and nGRIN fibers are again very small. This confirms that the discretization process does not change the propagation properties of our fibers.

**Figure 5:**
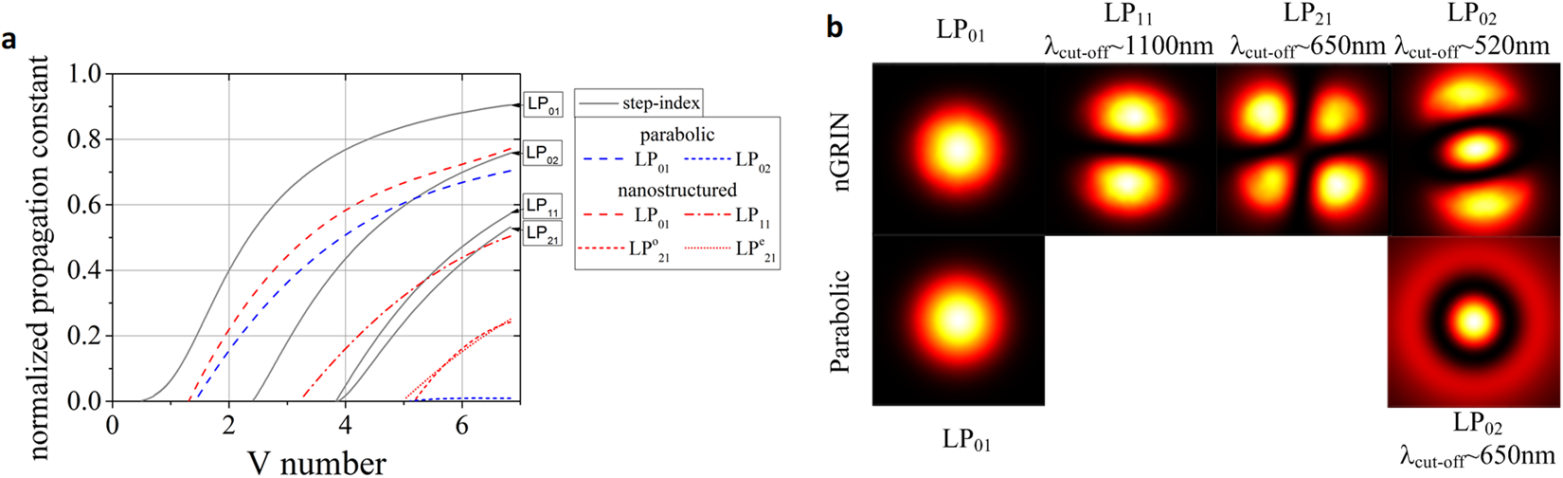
Propagation constants of modes supported by discussed fibers. (**a**) Normalized propagation constants for the *LP*_*mn*_ modes for fibers with various index profiles. Superscripts *o* and *e* denote polarization modes. (**b**) Intensity distributions of the modes for nGRIN fiber and the fiber with parabolic index profile. Distributions are calculated close to cut-off wavelength of each mode, accordingly with the plot a.

We also investigated the properties of higher order modes supported by considered nanostructured gradient profile fibers and compared the results to the fibers with a step index and parabolic index profiles. The results of the normalized propagation constants versus normalized frequency are presented in Fig. 5a.

The obtained results indicate that in the case of the fundamental mode *LP*_01_, the propagation constant of the nanostructured fiber is very close to the fiber characterized by parabolic distribution of the refractive index. What is more, due to the hexagonal shape of the nanostructured fiber core, there are also modes of non-circular symmetry, such as the *LP*_11_, *LP*_21_ and *LP*_02_ modes (Fig. 5b), ordered according to their cut-off wavelength. For the non-symmetrical mode *LP*_11_ the cut-off wavelength is the longest and equals to 1100 nm. Further, the fiber is characterized by birefringence, which was shown in the case of the *LP*_21_ modes in Fig. 5a and is more particularly discussed in the section concerning the fiber characterization.

### Nanostructured GRIN fiber development

As a proof-of-concept we selected the fiber with a parabolic profile of the refractive index. The stack and draw method was used for preform assembly similarly to the one used for photonic crystal fibers development. The nanostructured fiber was stacked accordingly to the pattern shown in Fig. 3. We selected particular profile to verify how well we can control a drawing process toward obtaining a designed profile. Moreover it allow us to compare the fabricated fiber core profile with previously reported parabolic profile in soft glass nanostructured core fiber^5^.

**Figure 6:**
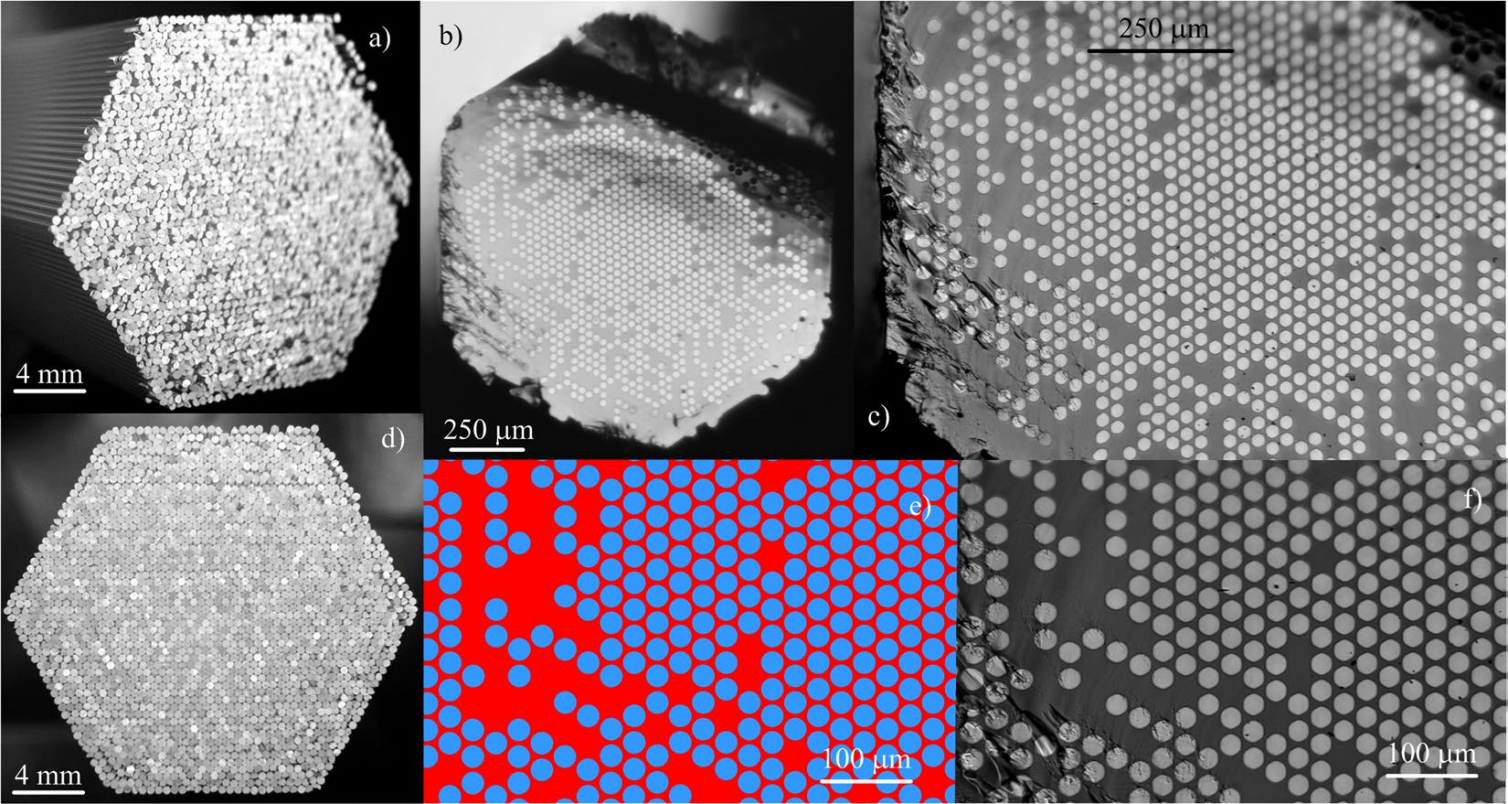
Stacked structural preform and subpreform on a facet of nGRIN element made of pure and Ge-doped fused silica. Structural preform presented in (**a**) side and in d front view. Each glass rod with diameter of 0.45 mm, can be distinguished in the preform. (**b**) SEM image showing cross-section of drawn subpreform of diagonal size 1.5 mm. (**c**,**f**) Zoomed subpreform view. Dark areas correspond to fused silica glass, while bright ones represent highly doped core (8.5% mol *GeO*_2_) of step index base preform. (**e**,**f**) The mosaic pattern of fiber design correspond to the subpreform structure.

The hexagonal structural preform was composed of 2107 glass rods with 53 rods on the diagonal, Fig. 6. The rods of low refractive index were made of fused silica Ohara SK1310 glass, while the rods with the high index value were made of a step-index preform from OptaCore. The core of the preform was Ge-doped (8.5% mol) fused silica, while the cladding was F300 fused silica from Heraeus. Simple geometrical calculations indicate that the effective concentration of germanium in a rod made of step-index preform is 4.9% mol. As the rod is scaled down to subwavelength size, it can be treated this way. What is more, due to diffusion, the Ge dopant will spread out into the silica cladding and the maximum concentration will be smaller than the initial one. We selected a step-index preform instead of Ge-doped rod for controllable dopant spread and to avoid grinding procedures and additional processing, in order to minimize roughness. The maximum concentration of our gradient fiber is comparable to one in a core of SM fiber, which is around 3% mol. Specific parameters of used glasses and the base preforms are listed in Table 1.

**Table 1:** Properties of the used glasses and glass preforms.

Parameter	Glass type		
	low index	step-index preform	
		core	cladding
	SK1310	Ge-doped F300	F300
Refractive index, *n*_*e*_	1.4609	1.47207	1.46007
Initial diameter [mm]	N/A	18.4	24
GeO_2_ concentration [% mol]	0	8.5	0
Final rod diameter [mm]	0.45	0.45	
Effective GeO_2_ conc. [% mol]	0	4.9% mol	
Effective *n*_*e*_	1.4609	1.4678	

The structural preform was assembled manually with use of glass rods of 0.45 mm in diameter. This process is time consuming (about 40 h/preform of more than 2000 elements, Fig. 6). However in the future this task can be automated with robotic assembly.

**Figure 7:**
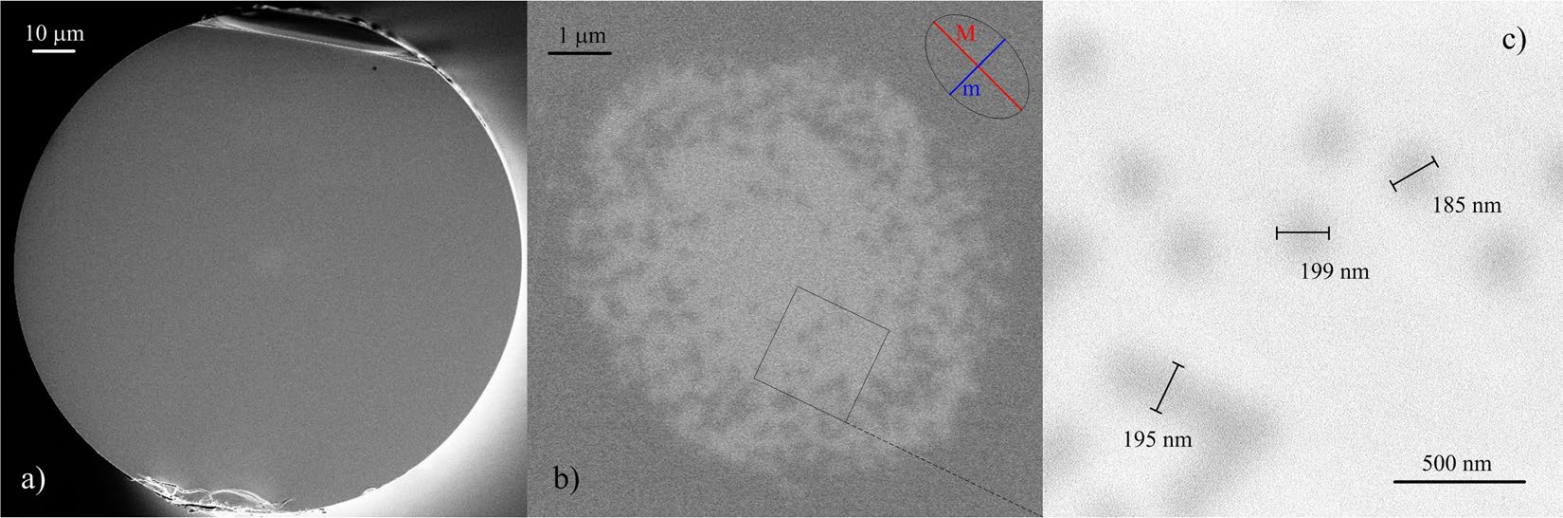
SEM image of drawn fiber #4. (**a**) Cross-section of fiber #4 with an elliptical core (**b**). Zoomed core area (**c**). Dark areas correspond to fused silica glass, bright ones are the Ge-doped silica rods.

The stacked preform was then fully thermally integrated and drawn into hexagonal subpreforms with diagonal dimensions in range from 1 to 3 mm, Fig. 6b,c,f. At this stage the bright circles can be distinguished in the subpreform cross-section. These circles correspond to the Ge-doped cores of step-index rods, which are distanced with pure silica claddings. As can be seen in Fig. 6(e,f) compared to scheme in Fig. 3, the designed structure was very well maintained in the drawn subpreform. Diameter of an individual rod for the 1.5 mm diagonal subpreform is about 28 µm. The subpreforms were then cladded with additional glass layers to achieve the required cladding diameter from 100 to 136 µm. Several fibers with various core and cladding sizes were fabricated. As an example, fiber #4 with the core diameter of around 8 µm and cladding diameter of 125.7 µm is presented in Fig. 7. The sharp borders between the fused silica claddings and Ge-doped cores are no longer distinguishable due to diffusion, however the mosaic structure is preserved. The diameter of individual rods in the core of this fiber is about 193 nm and was measured directly from SEM image fiber, Fig. 7c. Due to diffusion, the Ge-doped inclusion edges are blurred and the measurement uncertainty is as high as ±8 nm. According to the effective medium theory, the size of single inclusion fulfills the condition of λ/3 for the wavelength of 600 nm, while for longer wavelengths this condition is always better as for, i.e. 1550 nm it is λ/8. The designed pattern was calculated according to Fig. 3. and Eq. (5). This condition was limited to a hexagon circumscribed around the core, which was selected to simplify preform fabrication procedure. This limitation may cause appearance of modes with non-cylindrical symmetry.

Geometrical parameters of drawn fibers #1 to #5 are presented in Table 2. The cladding diameter is denoted as *ϕ*. The cores of fabricated fibers can be dimensioned with major (*M*) and minor (*m*) axis size and ellipticity (*η*), as in the case of elliptical core fibers. Fibers #2 and #3 have the cores closest in shape to the circle, which result in lowest ellipticity. Fiber #1, #4 and #5 became elliptical during drawing, which may in consequence cause residual birefringence in these fibers. The ellipticity of our fibers does not exceed 1.25, thus the birefringence is very small and can be neglected. This will be proved in one of the following sections of this paper. To check uniformity of drawn fibers the cladding diameter was monitored during drawing process and for all fibers the maximum cladding diameter uncertainty is 2%. Also the core dimensions were measured at few fiber sections distanced of tens of meters, showing the same 2% uncertainty. The fiber uniformity is then well maintained for hundreds of meters of a fiber.

**Table 2:** Geometrical parameters of fabricated fibers numbered from #1 to #5.

Fiber	*m* [µm]	*M* [µm]	*η*	*φ* [µm]
#1	6.51	7.82	1.20	100.2
#2	7.15	7.72	1.08	135.8
#3	7.44	7.88	1.06	103.7
#4	7.88	9.85	1.25	125.7
#5	8.41	9.72	1.16	124.4

### Nanostructured GRIN fiber EDS analysis

**Figure 8:**
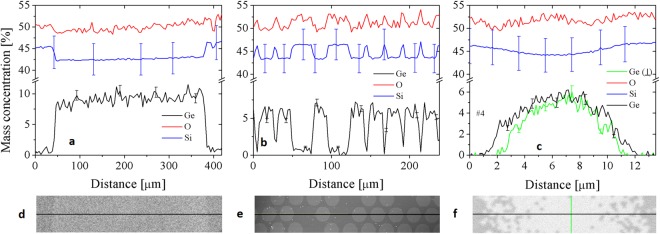
The chemical analysis of mass concentration of germanium, silicon and oxygen measured along the diameter of: (**a**) step-index rod, (**b**) fiber subpreform, and (**c**) final fiber #4 with corresponding SEM images (**d**–**f**).

In the previous section we have mentioned germanium diffusion, which occurs during the fiber drawing thermal processes. To analyze this effect, we have used the Energy Dispersive X-ray Spectrometry (EDS) and visualized the diffusion of germanium in a single step-index rod, in a fiber subpreform and in the final fibers. We took into consideration three elements: germanium Ge, silicon Si and oxygen O. We measured the mass concentration of these elements along diameter of a step-index rod, which was drawn in one thermal process from a step-index base preform (Fig. 8a,d). The average mass concentration of germanium in the doped area of the step-index rod is on the level of 9.2 ± 0.8 wt.%. The concentration of 0.12 ± 0.10 wt.% was measured in the area of undoped cladding. Obtained values are consistent with initial values for the step-index preform. The diffusion occurs on the core/cladding edge and, as was analyzed in^31^ for standard telecom fiber drawing, its reach is much smaller than 50 nm for one thermal process. Moreover Ge and Si concentration profiles complement each other in terms of the number of atoms, while oxygen profile follows silicon profile. The uncertainty of mass concentration of oxygen is very high (around 20%) and was excluded from the plots. In Fig. 8b the concentration profiles are shown for random row of Ge-doped inclusions of fiber subpreform, consequently after the second thermal process. The profiles of Ge and Si are again complementary and the concentration changes are consistent with SEM image in Fig. 8e. The average mass concentration of Ge in the centers of doped areas is now lower than for step-index rod and equals 6.95 ± 0.45 wt.%, while for undoped areas it is slightly higher as it is 0.32 ± 0.12 wt.%. The diffusion occurs, but each rod is still distinguishable in the SEM image and within the EDS analysis plots. After the third thermal process, which essentially was the final fiber drawing step, the effect of diffusion is the highest. The average maximum mass concentration in the center of fiber core is at the level of 5.4 ± 0.6 wt.%, while at the distance of 10 µm from the center this value is 0 wt.%. These values confirm concentrations estimated analytically, in respect to error range. The parabolic-like profile of Ge concentration distribution, measured along minor and major axis of #4 fiber core (Fig. 8c,f), is a result of Ge distribution and diffusion, and is identical with designed parabolic profile of index of refraction as its value depends only on Ge concentration, Eq. (4). The diffusion is very important in Ge-doped fiber drawing, especially for multi-step thermal processes. In such a composition of glass, it is mainly germanium, which spreads out due to diffusion. As a consequence, the diffusion effects can be predicted and diffusion processes can be controlled better, than it is in the case of multicomponent glasses^5^.

### Nanostructured GRIN fiber characterization

**Figure 9:**
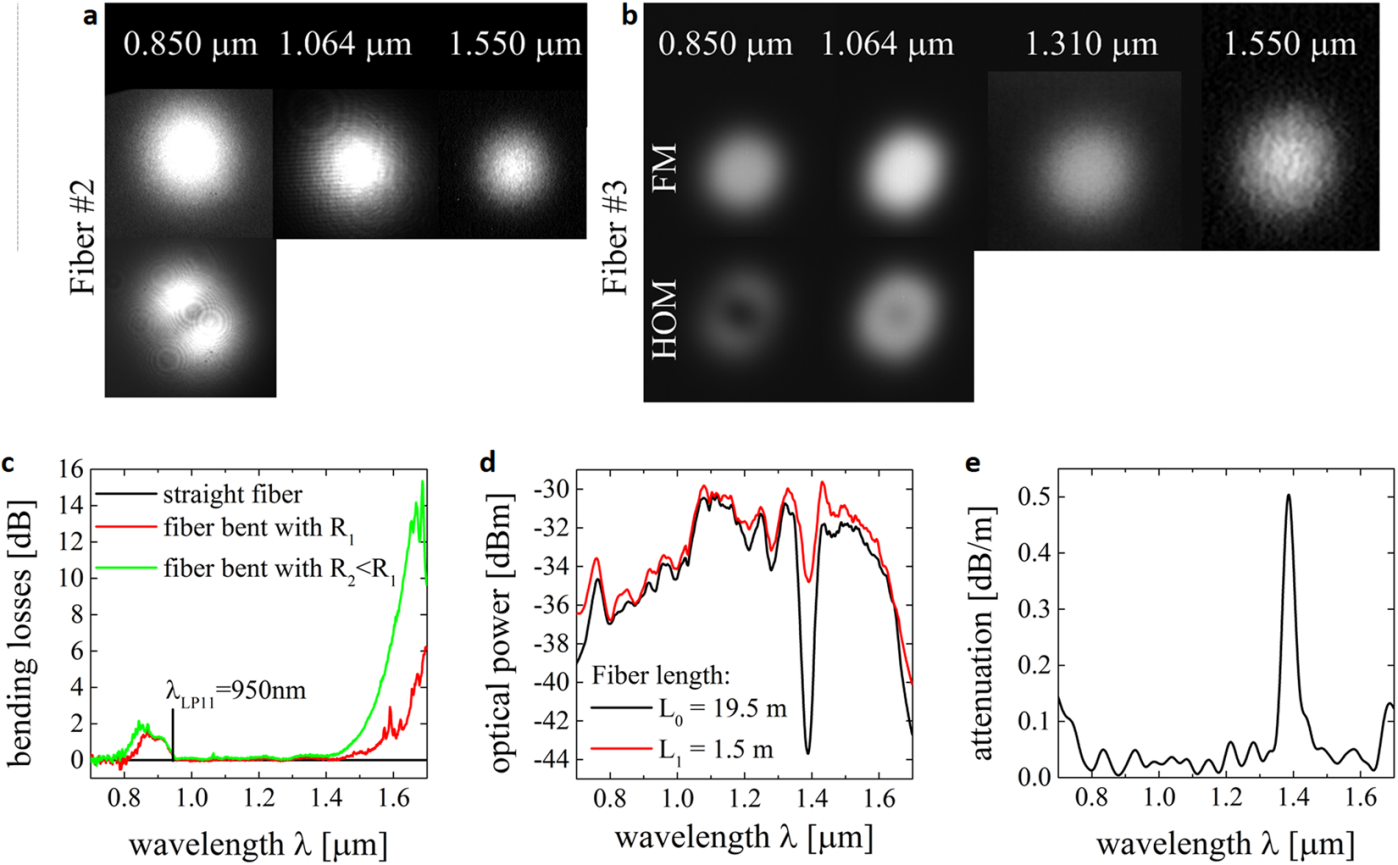
Cut-off wavelength and attenuation measurement. (**a**,**b**) Mode field distribution registered with the CCD camera at different wavelengths and for different coupling conditions for the fiber #2 and #3, respectively. (**c**) Bending losses characteristics showing cut-off wavelength of *LP*_11_ mode in fiber #2. (**d**) Optical power at the output of long and short section of fiber #3 and (**e**) calculated attenuation measured in broad spectral range.

Developed fibers were verified experimentally. At the first step the modal analysis was done by mode distribution measurements. Fibers #2 and #3 are still multimode at 0.85 µm and 1.064 µm and became single mode from 1.064 µm and 1.310 µm, respectively (Fig. 9(a,b)). This method is not critical for the cut-off wavelength (*λ*_*cut-off*_) estimation. In consequence *λ*_*cut-off*_ is between those wavelengths and additional measurements are required^32^. We performed this analysis for the fiber #2. The higher order modes (HOMs) were filtered from the output spectrum by bending (the fiber was protected with polymer coating). The losses generated in the bent fiber are plotted in Fig. 9c. Initially straight fiber was bent with radius *R*_1_ ~ 2 cm (red series) and with smaller radius of *R*_2_ ~ 1 cm (green series). Long-wavelength side of the attenuation peak placed closer to shorter wavelengths corresponded to cut-off wavelength for mode *LP*_11_ and equaled to λ_LP11_ = 0.95 µm. Similarly as it is for the step-index silica fiber bent with such a small radius (~1 cm) also fundamental mode (FM) suffers from bending losses, which could be seen for long wavelength edge of the investigated spectral range, where the attenuation increases very fast. The bending loss is at the level of 2 dB at 1.55 mm, which is a reasonable value for a laboratory fiber, as for commercial SM fiber it is about 0.5 dB^33^. The main reason for increased bending losses is lower difference between the effective index for the fundamental mode and the refractive index of cladding than in case of commercial SM fiber. As a result, the fundamental mode is less susceptible for confinement in the core during fiber bending. Bending losses can be reduced when refractive index profile is modified and effective refractive index of guided mode is increased. It is worth to notice that accordingly with the numerical simulations, the cut-off wavelength was at 1.1 µm for the fiber with the core diameter of 9 µm. As the fiber #3 has a smaller core, this cut-off wavelength does not exceed 1.1 µm. In the next step the numerical aperture *NA* was determined at 1.55 µm using a standard method and for all fibers it was determined at 0.11 ± 0.01. *NA* value is comparable to one of SM fiber, which equals to 0.14^33^.

The attenuation was measured using the standard cut-back method and in the considered spectral range from 0.7 to 1.7 µm it was at the same level of 0.05 dB/m for all fibers. Attenuation is only higher at the “water peak” located at a wavelength of around 1.4 µm, where it reaches 0.5 dB/m, Fig. 9e. The rods were prepared and stacked with high care under laboratory fume hood, to minimalize the pollutions in the stacked preform. What is more, the rods have quality for fiber drawing and are additionally flame polished before stacking, so the roughness of the rods is minimalized. Nevertheless, the losses of ‘research grade’ fibers are high and incomparable to those of commercial fibers. The reasons for losses are limited quality of germanium doped preform we used and limited cleanness of fiber stacking and drawing conditions, typical for research laboratory. The losses can be further reduced by use of high quality optical materials and use industrial standard of clean room for assembly of fiber preform and fiber drawing.

A part of nanostructured fibers developed in scope of this study had elliptical cores, which introduces residual birefringence. We have investigated the group birefringence with use of the wavelength scanning method^34^ for long sections (18 m) of fibers #2, #3 and #5. We have selected fibers #2 and #3 because of the lowest ellipticity of the core and the fiber #5, because of the ellipticity which is a consequence of the particular conditions of technological process. Linearly polarized light was coupled to the fiber #2 and analyzed at the output with a linear polarizer. The output signal was not sensitive for the changes of the polarization azimuth, even in a very long fiber section. The same behavior was observed for fiber #3. The fiber #5 was polarization sensitive and modulation of the output spectrum with the spectral interference fringes was recorded. The average spectral width of the fringes was 25 nm, for 18 m long fiber. The group birefringence was then estimated at (4.5 ± 0.5) × 10^−6^ for the wavelength of 1.55 µm. Group birefringence value is very low in fiber #5 and, as it is out of the measurement range, is much lower for fibers #2 and #3. For fibers #1 and #4 it is not expected to be considerably increased because of higher ellipticity of the core, but that was not measured. All presented fibers have residual birefringence, which corresponds to the appearance of asymmetric modes *LP*_11_, and this was in agreement with numerical simulations. As the birefringence is at the level of 10^−6^, it could be neglected.

**Figure 10:**
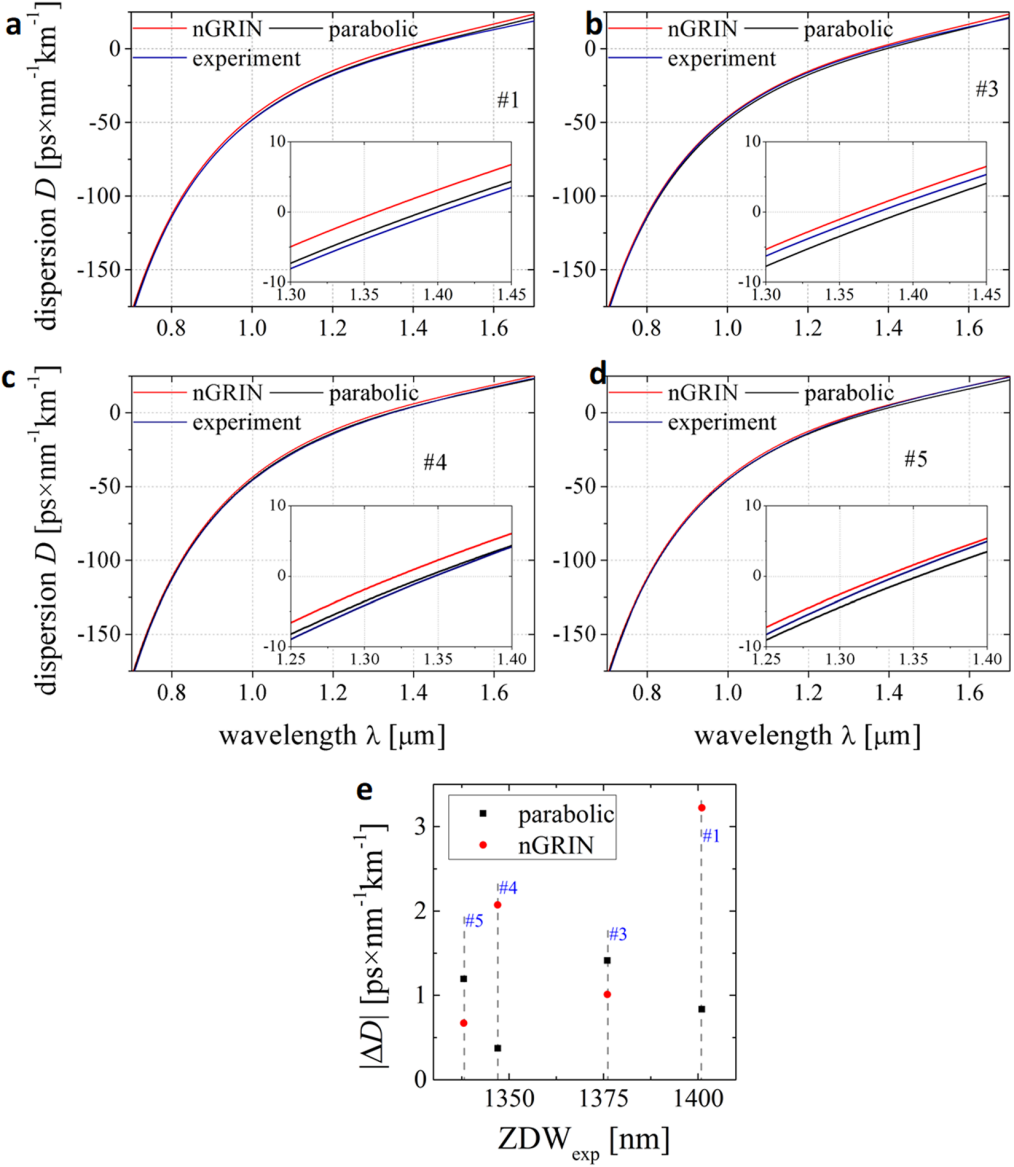
Measured and calculated characteristics of dispersion *D* for nanostructured fibers (**a**) #1, (**b**) #3, (**c**) #4 and (**d**) #5 and (**e**) dispersion difference Δ*D* in a function of experimentally estimated ZDW.

We also investigated the dispersion *D* of our fibers. Measurements were conducted with a Mach-Zehnder interferometer with full compensation of optical elements in both arms^35^. We have also numerically analyzed the dispersion of fabricated fibers. The structures were implemented in a numerical model assuming the subpreform pattern (Fig. 6b) and core sizes of final fibers (Fig. 7d). Simulations were also performed for the exemplary fibers with parabolic refractive index distribution and hexagonal shape of the core with diagonal core size corresponding to average core size of real fibers. The ellipticity of the cores was neglected. The dispersion characteristics measured and calculated are plotted in Fig. 10(a–d), separately for fibers #1, #3, #4 and #5. The differences in *D* value close to experimentally estimated ZDW are plotted in Fig. 10e.

The *D* parameters calculated for real structures are plotted as red series named “nGRIN” in Fig. 10. The experimental results are plotted as blue series. We also calculated the parabolic (black) distributions assuming the averaged size of the cores, respectively for each fiber. The nanostructurization with arbitrary parabolic index distribution with the inclusion size of λ/3 results in index distribution very close to the parabolic shape, what is numerically confirmed for the investigated fibers. The experimental results allow to verify that the dispersion shape is in agreement with results of the numerical simulations. Small discrepancies in dispersion parameter *D*, not exceeding ±3.5 ps × nm^−1^ km^−1^, can be assigned to measurement uncertainty, Fig. 10e. The Δ*D* values correspond to mismatch between measured and calculated zero dispersion wavelengths. If we consider the dispersion differences between experiment and ideal (parabolic) distribution Δ*D* value is lower than ±1.5 ps × nm^−1^ km^−1^.

Summarizing, the dispersion characteristics obtained experimentally match the predicted profiles very well. It again confirms that the drawing process is well controlled and the method allows development of fibers with predicted parameters. Previously^5^ we have shown that for the soft glass we could not control ion distribution during drawing, and as a result the developed fibers had very different characteristics than designed with numerical simulations. This was related to nonuniform diffusion of various ions in the used glasses. Currently, in case of Ge-doped silica glass, we can consider only two types of ions, Ge and Si. Since their size and molecular number are relatively close, their mobility in glass is also similar during the drawing at temperatures near or above the softening point.

## Discussion

We developed a proof-of-concept graded index fiber with nanostructured core based on fused silica glass. Graded index profile of the fiber core is obtained with arbitrary distribution of more than two thousand rods made of silica and Ge-doped silica with diameter of about 193 nm each.

Effective refractive index distribution in drawn fibers with nanostructured cores is similar to the predicted continuous graded index profile, which was verified by EDS analysis. We also confirmed the performance of the fibers by means of dispersion measurement, since it is very sensitive for refractive index changes. Obtained values of dispersion are precisely reproduced by numerical simulations as the differences between measured and predicted dispersion characteristics at ZDWs do not exceed ±1.5 ps × nm^−1^ km^−1^. We have also verified experimentally that developed fibers are single mode above 1.1 μm. The small ellipticity of the core, which results from drawing conditions, has negligible influence on optical properties of the fibers as the residual birefringence is at the level of 10^−6^. The attenuation is 0.05 dB/m, which is low for laboratory grade fiber. It confirms that no additional pollutions were introduced to the preform during the technological processes of nanostructurization. It also proves that with this method we can control the drawing process and develop fibers with arbitrary index distributions in the core and that we are able to shape the optical properties with significant engineering freedom.

We proved that the nanostructurization idea, previously applied to design and fabricate borosilicate fibers^5^, works properly for fused silica fibers and allow design a fiber with specified parameters and develop it strictly according to the design. Fabrication of a single mode fibers with dispersion engineered profiles and multimode fibers with engineered group velocity for different guided modes can be then realized with the proposed method. What is more, it is possible to develop the fibers with non-radial symmetry, i.e. rectangular shaped mode fibers are highly demanded for laser ablation applications. What is more, fibers with artificially anisotropic glass core can be manufactured with nanostructured approach, as well. None of the fiber types mentioned above could be developed with standard technology for fused silica fiber manufacturing.

## Methods

### EDS analysis

**Figure 11:**
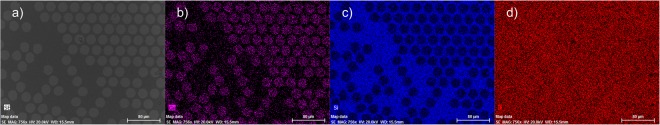
The SEM image of zoomed fiber subpreform and the chemical composition of the same subpreform area.

Energy Dispersive X-ray Spectrometry was performed with the use of high resolution Scanning Electron Microscope (Zeiss). The analyzed samples were: step-index rod, fiber subpreform and final fibers from #1 to #5. For better conductivity the thin layer of about 6 nm of coal was deposited on all samples. The chemical analysis was performed for germanium, oxygen, silica and coal. To avoid confusion of spectroscopy lines characteristic for elements that are less heavy than coal, the convolution mode was used. The procedure enables to deconvolute spectroscopy signal of coal from the measurement and give more precise results for germanium, oxygen and silica. The investigations were conducted in two ways. First was a quantitative analysis, giving value of mass concentration in point or frame placed in the center of doped or undoped area of the sample. Second was profile analysis along diameter or principal axis of the sample, in which the atoms of interesting element were counted. The results were combined together to express measured profiles in mass concentration values instead of atom counts (Fig. 8). The third way for chemical analysis is to create the map of elements distribution, which represents analyzed area of a sample in the atoms counts for element. The results are shown in Fig. 11: **a** SEM image of zoomed subpreform with element distribution maps for **b** germanium, **c** silica and **d** oxygen.

### Fiber fabrication

The glass rods preparation: pure fused silica glass (SK1310) was formed into a glass rods of diameter of 0.45 mm. Ge-doped step-index preform (OptaCore) was also drawn into glass rods of the same diameter. Then the structural preform was stacked of the glass rods layer by layer, accordingly with the calculated pattern from Fig. 3. 2107 glass rods were used and the final element had 53 rods on the diagonal. Preform was welded and drawn into subpreforms of diameters ranging from 1 to 3 mm. Subpreforms were then cladded with additional glass tubes and filled with glass rods of pure fused silica glass to draw final fibers with demanded diameter and nanostructured central core area. All drawing processes were conducted using drawing tower typical for fused silica fibers manufacture. Typical technological parameters of fiber drawing were as follows: preform diameter: 16.5 mm, preform feeding speed: 0.25 mm × min^−1^, drawing temperature: 1960 °C, drawing speed: 4.2 m × min^−1^ for a fiber with diameter of 135 μm and 5 m × min^−1^ for a fiber with *ϕ* = 125 μm. Fiber #2 was additionally protected with polymer coating to investigate cut-off wavelength with bending procedure.

### Fiber proceeding

**Figure 12:**
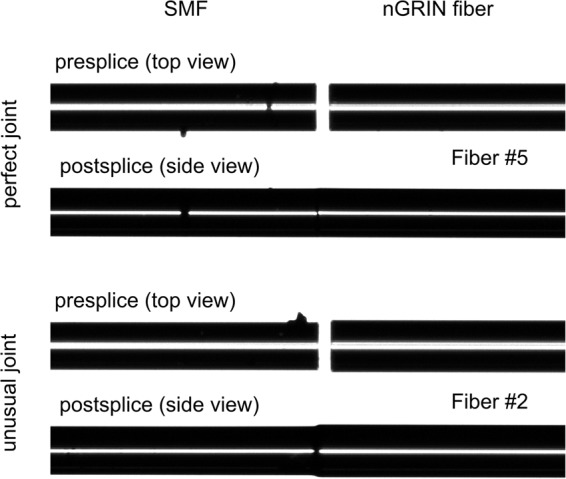
Splicing test results for perfect and unusual joint of nGRIN fiber with hSMF.

The cutting method of nGRIN fiber is standard as for all-solid silica fibers. The splicing procedure is exactly the same as for telecom fibers. Typical SM to SM splice program was used for fiber joint. In Fig. 12 the results of splicing nGRIN fiber to SM fiber is reported: the nGRIN fiber with compatible core and cladding diameter (perfect joint) and the nGRIN fiber with compatible core and larger cladding diameter (unusual joint). The average splice loss for perfect joints was at the level of 0.26 ± 0.07 dB, which is similar to typical value, which is 0.05 ÷ 0.2 dB. The losses for unusual joint were much higher, as the fiber diameters were mismatched.

### Fiber characterization

Modal analysis was conducted using two methods: transmission measurement of bent fiber and by imaging of the mode intensity distribution. In the first approach only fiber #2 was investigated, because of lack of the polymer coating in other fibers. The transmission was measured using broadband (600–1700 nm) optical spectrum analyzer for straight and for bent fiber. Bending allows to cut from the transmission spectrum the intensity of higher order modes supported by the fiber at the cut-off wavelengths of each mode. In the second approach the mode intensity distributions for all fibers were captured using two CCD cameras: with silicon sensor and with additional phosphor layer for mode inspection around 1.55 μm. The mode intensities were recorded for different wavelengths and for different coupling conditions. For numerical aperture measurement the mode intensity distribution was also recorded in a function of distance of fiber facet measured in respect to the CCD sensor. This allows to calculate the Gaussian mode size in a function of the distance from the fiber facet. The dispersion was measured in Mach-Zehnder interferometer by recording equalization wavelength for each compensation length of the interferometer. Group birefringence was measured using the wavelength scanning method. Attenuation was measured using cut back method.

### Verification of fiber uniformity

**Figure 13:**
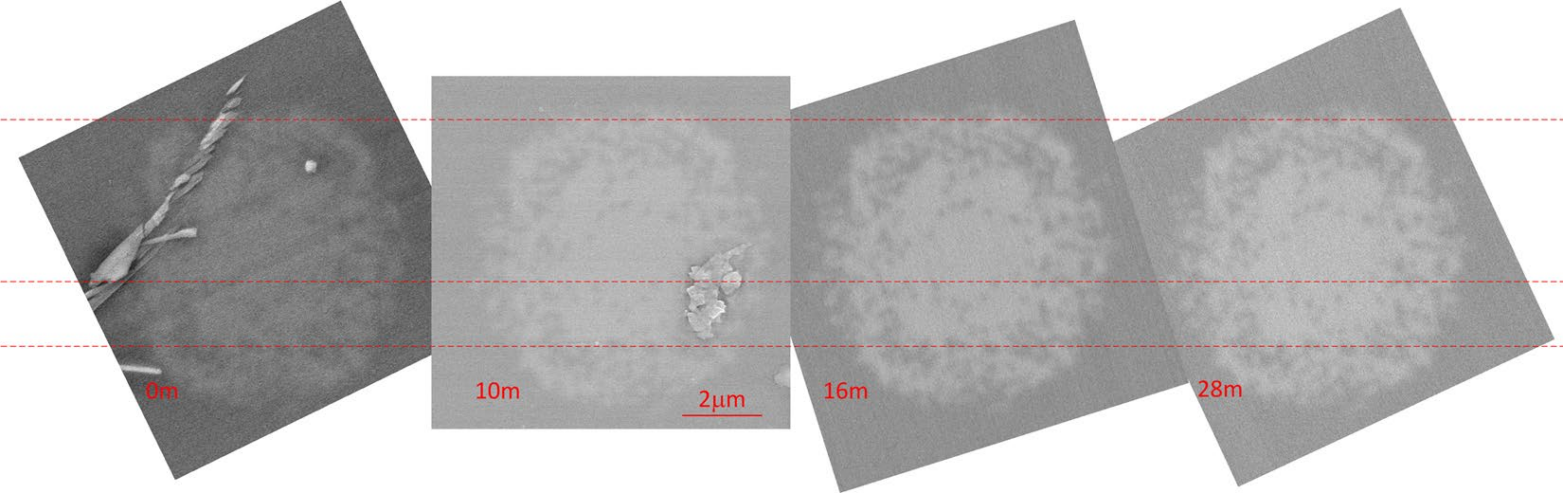
SEM images of cross-sections taken at “zero” position (0 m), 10^th^, 16^th^ and at 28^th^ meter of a fiber #2.

Scanning Electron Microscope (Zeiss) was used to capture the series of images of fibers cross-section collected at different length of fibers. The fibers were firstly covered with thin (6 nm) layer of carbon to enhance its conductivity. Fibers #1 to #5 were cut with standard cleaver and at least 3 probes of each fiber were investigated. To analyze the uniformity of the drawn fibers the images were also taken for the fiber cross-sections at four random points at a distance of about 28 meters. The results are presented in Fig. 13 for the fiber #2. The first picture was taken after fiber drawing, so at a point “zero” (0 m). Subsequent images were taken for cross sections at 10^th^, 16^th^ and 28^th^ meter of a fiber. Red dash lines are crossing the nanostructure pattern in characteristic places for better comparison. As it is seen, the structure uniformity is preserved, which is a consequence of all-solid design. The averaged values of dimeters of fiber cladding *ϕ*_avg_ and core *mM*_avg_ with its uncertainties are gathered in Table 3. Averaging means that the dimensions measured along minor and major axis of a fiber are averaged neglecting the ellipticity. In general all dimensions have the uncertainty smaller than 0.5%. The technological diameter deviation along the fiber is 1.63 μm, which is about 1.2%. The dimensions deviation of 1.2% stands also for the core size. The deviation of inclusions was not investigated, as the measurement uncertainty was as high as 4%.

**Table 3:** Cladding and core diameter uniformity along the fiber #2.

Distance in a fiber #2	*φ*_avg_ [µm]	Δ*φ*_avg_ [µm]	Δ*φ*_avg_ [%]	*mM*_avg_ [µm]	Δ*mM*_avg_ [µm]	Δ*mM*_avg_ [%]
0 m	135.80	0.43	0.32	7.44	0.032	0.43
10 m	135.30	0.38	0.28	7.41	0.029	0.39
16 m	136.05	0.34	0.25	7.45	0.027	0.36
28 m	136.93	0.41	0.30	7.50	0.031	0.41

### Data availability

The data that support the findings of this study are available from the corresponding authors upon request.
